# Organizing Health Care Networks: Balancing Markets, Government and Civil Society

**DOI:** 10.5334/ijic.3960

**Published:** 2018-07-11

**Authors:** Kasper Raus, Eric Mortier, Kristof Eeckloo

**Affiliations:** 1 Ghent University Hospital, Ghent, BE; 2 Department of Philosophy and Moral Sciences, Ghent University, Ghent, BE; 3 Faculty of Medicine and Health Sciences, Ghent University, Ghent, BE; 4 Faculty of Medicine and Health Sciences, Department of Public Health, Ghent University, Ghent, BE

**Keywords:** health care networks, markets, government, civil society, ethics

## Abstract

Much is changing in health care organization today. A perspective or paradigm that is gaining ever increasing momentum is that of translational, extramural and integrated care. Current research suggests many potential benefits for integrated care and health care networks but the ethical issues are less frequently emphasized. Showing that integrated care can be beneficial, does not mean it is automatically *ethically* justified. We will argue for three ethical requirements such health care networks should meet. Subsequently we will look at the mechanisms driving the formation of networks and examine how these can cause networks to meet or fail to meet these ethical requirements or obligations. The three mechanisms we will examine are government, civil society and market mechanisms, which, we argue, should be balanced properly. Each mechanism is able to provide a relevant ethical perspective to health care networks. However, when the balance is skewed towards a single mechanism, health care networks might fail to promote one or more of the ethical requirements.

## Introduction

Much is changing in health care organization today. A perspective or paradigm that is gaining ever increasing momentum is that of translational, extramural and integrated care [1,2,3]. It is being acknowledged that tackling health problems within a particular area or community and providing proper care for individual patients (with often diverse and complex needs) requires the integration of multiple services, professions, and organizations [4]. It is clear that helping communities and patients requires one to look beyond the walls of particular institutions or organizations.

Integrated care not only requires interprofessional collaboration, but also interorganisational integration, cooperation and/or collaboration [5]. This often takes the form of health care networks (HCNs), which are rapidly becoming increasingly common [6,7,8]. Many terms or concepts can be used to refer to such collaborations, but for sake of clarity we will continue to use the concept of ‘health care network’. There are two reasons for doing so. First, we prefer ‘health care’ to ‘hospital’ or ‘clinical’ as it makes clear that such a network can encompass *all* aspects of health care; from highly specialised treatments to basic care. Second, we prefer ‘network’ to ‘alliance’, ‘collaboration’ or ‘cooperation’ as it better emphasises the interconnectedness of the various health care organisations and the multiple and often complex interactions between the members of that network. A network is not automatically associated with a particular form or governance model [9,10,11]. In order to cover as many forms of networks as possible, we will use the broad and well-known definition of a network as *‘groups of three or more legally autonomous organizations that work together to achieve not only their own goals but also a collective goal’* [11].

The goal of integrated care and HCNs is to enhance the performance of the overall health care systems in terms of economic efficiency, quality, innovation, patient satisfaction, etc. Several empirical studies indeed suggest that HCNs might enhance efficiency and promote quality [12,13]. However, the challenges remain numerous. There is, for example, discussion on how ‘integrated care’ and ‘collaboration’ should be understood [14]. As a result, it is highly difficult to determine when integration or collaboration is successful. A recent systematic demonstrated the existence of no less than 144 tools for measuring progress towards integrated care [15]. There is also evidence that despite the reported successes, a substantial number of HCNs fail [16]. There is currently argued to be only limited insight into why this is so [16,17] and one must therefore remain careful not to consider them a panacea.

Moreover, it is important not to lose track of the ethical justification for such integrated care and broad HCNs. The fact that integration and collaboration can be beneficial does not mean it is automatically *ethically* justified in any form or under any circumstance. An economically efficient HCN could result in a highly unfair allocation of healthcare. Therefore, not only do we need more insight into the workings of such HCNs, there is an urgent need to consider more ethical issues. In this paper we identify some ethical obligations we can expect them to meet, regardless of their form. Subsequently we will look at three mechanisms influencing and coordinating the formation of HCNs and examine how these can cause such networks to meet or fail to meet these ethical conditions.

## Ethical principles for health care networks

Ethically speaking collaboration and HCNs should be justified based on a *precautionary principle*. When faced with significant and potentially harmful changes, the burden of proof should lie among those in favour of these changes. This could be argued to be the case for integrated care and HCN which would involve drastic systemic changes within the health care system, but where it is still unsure what causes success and what causes failure.

Which ethical requirements we can expect HCNs to meet is debateable, but the well-known and often used principlist framework of Beauchamp & Childress’ [18] could provide a good starting point. Using this framework, we believe it is possible to deduce at least three categories of ethical requirements or obligations.^1^ Off course, this should in no way be taken to be an exhaustive list. For this paper we have limited ourselves to those obligations or requirements that are, we believe, both basic and widely acceptable.

First, in line with the principles of integrated care [19], HCNs must result in an equitable and **just** provision of medical care. They should operate according to principles of distributive justice that are applied reasonably, transparently, consistently, and coherently. Organising HCNs so that they lead to health care being distributed in an arbitrary way or according to criteria that are accepted to be morally irrelevant, clearly fails to meet principles of justice.

Second, HCNs must at least **not be harmful** for relevant parties and should, preferably, even be **beneficial** when compared to the current situation. A significant amount of research indicates that there may be many advantages to HCNs [13,20,21,22]. However, this does not mean this the case for every HCN. Also, there are also potential harms. HCNs may, for example, result in harmful diffusion of responsibility and have been shown to potentially result in increase in prices for medical services [23].

Third, HCNs must not unduly interfere with patients’ (and other parties’) **autonomy**. There should be an ethical default in favour of allowing people to make autonomous health related choices based on a plurality of different values and interests. HCNs can thus be ethically evaluated based on the degree to which they respect and promote such choices.

We will argue that meeting these principles requires a proper balance between three mechanisms driving or influencing the trend towards integrated care and the formation of HCNs.

## Mechanisms of coordination (government, markets, and civil society)

What drives collaboration, integrated care and HCNs is likely to be as diverse as the number of forms it can take. Here we will focus on three mechanisms. First, health care institutions can come to form HCNs in response to (*governmental*) policy to promote or even enforce such networks. Second, collaboration in networks can be driven or influenced by economic *market-based* considerations. Third, HCNs can be formed voluntarily when health care institutions within *civil society* come together around shared goals, values and/or interests [24,25]. In this paper we will focus on the government, market and civil society triplet as they effectively capture the political, the economic, and the social drivers of HCNs. We will show below that these forces are indeed at work.

We acknowledge that alternative sets of mechanisms might be formulated [26]. However the distinction between government, markets, and civil society will be used in this paper as it is common and insightful. Also, our triplet is akin to the widely used ‘hierarchy, market and networks’ triad first formulated by Powell (1990) [27]. Governmental policy is a hierarchical way of organization where governmental agencies enact policies enforcing or promoting integration, collaboration and cooperation. ‘Networks’ refers to the more voluntary collaboration of institutions based on reciprocity and shared interests. As such, it accords to our use of ‘civil society’.

### Government

There is a clear policy push towards integrated care and health care networks [3]. HCNs have been implemented, encouraged and/or embraced as a policy measure by governments (e.g. the NHS Clinical Networks). Various case studies indeed suggest that governmental policy can operate as an important catalyst for HCN formation [28,29]. Another relevant example of such policy is Belgium, where the government is developing policy that would legally require all Belgian hospitals to become part of a single larger hospital network. This HCN must provide almost the entire continuum of hospital care (secondary, tertiary *and* quaternary) [30]. Although initially intended as *hospital* networks, these HCNs are explicitly intended to offer a platform for more intensive and better communication with primary care and should thus function as a first stepping stone towards more fully integrated health care. Norway and Denmark similarly has a governmental policy to promote interorganizational coordination in health care [31,32].

### Markets

Second, economic considerations drive changes in the organisation of health care, often in the shape of market thinking (supply/demand logic, deregulation, privatisation, and liberalisation). Different health care institutions or networks often provide the same services and patients are free to choose between institutions or networks. This naturally forms the basis for some degree of market thinking and economic competition, both nationally and internationally. Within the European Union, for example, the right to receive treatment across the EU is guaranteed by the European Directive on Cross Border Health Care.

In many HCNs today, market mechanisms are indeed at play. A 2013 American study showed how economic forces drive the creation of locally integrated health systems [23]. Another example is the more and more common phenomenon of hospital acquisitions where a larger health care system acquire smaller health care institutions [33].

### Civil society

Third, a significant role might be played by civil society, which refers to ‘the space for collective action around shared interests, purposes and values, generally distinct from government and commercial for-profit actors’ [20]. Within this space social entities (groups, institutions, associations, etc.) that share interests, values or ideology can come to collaborate and form HCNs. Such so called ‘emerging self-organizing behaviour’ and coming together of like-minded institutions in the health care domain is inevitable and an important driver towards integrated care. Having a shared mission is recognized as being one of the key factors in determining a network’s chance of success [17].

## Justification for a proper balance

### Relevant ethical perspective provided by government, markets, and civil society

Democratic governments have an obligation to further the interest of all citizens regardless of personal or political convictions. Hence when it comes to the organisation of care and the allocation of medical goods within HCNs, they are able to stress the importance that care is allocated in a way that is **just and fair** for *all*. Governments focus on patients as equal **citizens** with equal *basic rights* to good quality health care. In this way, governments are able to emphasise and, if necessary, enforce a relevant aspect of patients that is needed to achieve full patient centeredness.

Second, market thinking could provide integrated care and networks with the required **efficiency**. In a context where scarcity is an undeniable reality, efficiency operates as ethical principles making sure resources are used or allocated with a minimal amount of waste. A health system or HCN that does not allocate resources efficiently fails to do the most good they can do, thereby failing to fulfil their ethical duty of beneficence. Moreover, a supply and demand model can be an additional assurance that HCNs are responsive to **the actual demands of patients**. In this way markets hereby emphasize a different but equally relevant aspect of patients. It conceives of patients as **consumers** with particular wishes, demands and expectations.

Third, civil society is able to provide for different networks and a **plurality** of different **values** according to which care could be organised. The various agents within civil society voluntarily collaborate and can form HCNs around common goals and values in order to collectively achieve them. This plurality of values can help guarantee **autonomous choice** for patients and caregivers who are able to choose the care or HCN that accords best with their own values. Collective action in the civil society space emphasises the patient as part of a (geographical, ideological, political or social) **community**.

In short, government can guarantee HCNs respect patients’ *rights*, markets guarantee they respect patients’ *demands* and civil society guarantees they respect patients’ *needs and values*. Together they provide integrated care and HCNs a necessary all-encompassing ethical justification. If, however, the balance is skewed, there are potential unethical consequences.

### Unethical consequences of a skewed balance

#### Government

Integrated care often has high symbolic value [34], but might best be considered a means to an end (increasing the performance of a health care system) rather than a goal in itself. Matters of distributive justice must be primary when considering the organization of HCNs. Although there is debate about whether health care is a public or private good, it is clearly a good that is both *basic* and of *common interest*. Like, for example, law enforcement and education, organised health care belongs to the basic fabric of society. Through good health, citizens are able to participate fully in society, making the provision of health care a democratic duty.

Of course, there remains considerable debate as to what constitutes the best ethical criterion for guaranteeing justice and fairness in health care [35]. According to egalitarian theories of distributive justice, the goal should be to create an equal distribution, whereas others emphasise that distribution should happen according to medical need. However, even though there is debate on what amounts to a fair criterion of distributive justice, there is likely to be considerable consensus on what qualifies as an unfair criterion. For example, allocation of medical resources based on discriminatory criteria such as skin-color, gender or sexual orientation are clearly unjust. Governments have an ethical duty to continuously monitor whether integration or collaboration leads to unfair allocation and to respond if necessary. As such, government involvement provides the necessary checks and balances.

HCNs could result in unfair allocation. For example, in 2011 the US the Carolinas HealthCare System Levine Cancer Institute covered 38 hospitals and all patients had to travel to a central hospital for specialized cancer care. Such a structure benefits those living close to the central hospital or those able to travel, which could be unfair. In response, the network switched strategy and created more than 20 decentralized centers and clinics for cancer care [6]. In order to guarantee fairness Belgium – in its current policy on HCNs – will require every network to provide or guarantee almost the entire spectrum of medical care in a fair geographic distribution [30].

However, when governmental influence is too strong, there are also potential risks. First, HCNs may become overly political and might become overly formalised and rigid. It has been argued that governments often favour a more classic bureaucratic hierarchical arrangement, whereas networks are characterised by more complex and less hierarchical structures [36]. In order to maximize the benefits of the network mechanism, sufficient room for the network to more autonomously develop its own organisation and governance is required.

Second, using policy to fully mandate institutions to collaborate in a network might not work. Research indicates that trust and leadership are important conditions determining a network’s success [16,17] and these cannot be simply enforced. A case study from Canada provides a telling example of how mandated collaboration can result in a failed network [34].

Third, there is a risk that governments enforce their own values, thereby ignoring ‘the fact of pluralism’ [37]. Governments represent all citizens, and not just their own view or the majority view. When governments force certain values upon HCNs or only accept networks that avow a particular value, the ethical principle of both patient autonomy and institutional autonomy is not respected. One example is that of a Canadian child health network which worked, successfully, in close collaboration with the provincial department of Health Staff. However, it was also reported that ‘at times the provincial staff tried to control the Network strategic agenda and micromanaged key shared projects’ [29]. A respondent reported that this ‘really caused a lot of problems’ [29].

#### Markets

Many people strongly believe that a free market mechanism can help organize medical resources most efficiently and can increase quality of care and some studies seem to confirm this [38]. If markets are absent to drive integrated care, the argument goes, integration and HCNs are not in accord with economic realities and are not sustainable in the long run.

However, despite the potentially good effects of markets as a way to allocate scarce good, there are several ethical risks when market thinking becomes overly dominant [39,40]. First, from an ethical point of view, markets can only operate properly when several background conditions are met. For example, fair markets require the actors operating in that market to behave as **equal** and **rational** consumers. Ideally, this would allow fair competition between hospitals or HCNs and promote quality while driving down prices, as patients would seek out hospitals or networks that provide the best service at the lowest cost or might be willing to pay more for a better medical service. However, it is clear that most patients are not perfectly rational market oriented consumers as they are relatively unaware of differences in quality or price between hospitals or HCNs. Even when they are aware of the difference, it is often difficult if not impossible for them to reliably assess quality of medical care in a particular hospital or HCN beforehand. Rather, patients’ choice for a certain hospital is often driven by chance (e.g. where an ambulance takes you), geography (e.g. the only hospital in the region where you live) or personal connection (e.g. the hospital where you or your family always go, where you know someone or where you had a previous positive experience) [41]. Requiring patients to be rational consumers might also be morally problematic if it is employed as a technique to displace an institution’s duty and responsibility to provide quality care towards a patient responsibility to choose the best HCN. This is why in integrated care there is often argued to be a need for case management where individual patients are guided through the complexity of the networks towards the best care [42].

Moreover, even if patients were *rational* consumers, they are not *equal* consumers. It might not be possible for certain patients to travel to (or even within) the HCN that provides the best or cheapest care, for example because these patients are too sick or do not have the money to travel. This would mean that those patients who are economically advantaged or fortunate to be able to travel, can maximally profit from mere market working. This could hardly be said to constitute a fair or just way to allocate goods.

Second, HCNs provide a common good, namely health or care, which might be corrupted when it is bought and sold on a free market, thereby becoming a mere tradeable good. A potential risk of such an evolution is that the networks move away from what patients need and divert to providing only care that is profitable. A study in the US shows, for example, that for-profit hospitals are more likely to offer profitable services while not providing unprofitable services [43]. Other studies indicate how close collaboration and mergers of hospitals has led to increases in prices [23]. Large HCNs could perhaps generate full or quasi monopolies within certain market segments of geographical areas. This brings along the risk that the commodification of health care resources becomes more structural, systematic and thus less avoidable for patients.

Also, somewhat paradoxically, in the specific case of HCNs, dominant economic incentives might on the one hand drive collaboration while, on the other hand, undermining the success of that collaboration. Market mechanisms might influence the way in which a network itself is organised and it might even result in competition between the partners of the network.

#### Civil society

Within the civil society institutions with particular values and interests, can come to collaborate to form HCNs. In this way HCNs can be responsive to the needs and values that exist within a certain community. This in turn increases patients’ freedom and autonomy as they are able to balance economic and non-economic values when choosing networks.

When coordination through civil society actors is absent, HCNs are at risk of being bereft of value and of becoming mere market based entities or formal and value-less instruments of political constellations. There is also evidence to suggest that taking away self-organization of care might threaten the stability of networks as shared values and an agreement on the importance of the shared mission is a factor determining HCNs’ success [16,17].

However, if civil society is overly dominant, this could lead to a loss of an overarching perspective to integrate and streamline the different projects. Organizational choices made by various HCNs (for example, to use a certain way of allocating resources, to not provide certain services or to not perform certain practices (e.g. abortion or euthanasia)) can have significant implications on the fairness of the overall health care system. If actors within civil society are completely free to organize themselves in any way they see fit and according to whatever values they prefer, neither basic care nor justice might be guaranteed. This, for example occurred in Bangladesh. Schurmann & Mahmud analysed the role of civil society in organising health care in Bangladesh and found that ‘with a few exception, civil society in Bangladesh replicates the structural inequalities of society at large’ [44].

## Concluding remarks

Integrated care and interorganisational collaboration is widely praised, but still needs to be justified from an ethical perspective. We have argued that (1) they must guarantee a fair and just provision of medical care, (2) they must be maximally beneficial and minimally harmful, and (3) must not unduly interfere with patient (and other parties’) autonomy.

For these three ethical obligations to be met a balance must be sought between three mechanisms driving the evolution towards HCNs: government; markets; and civil society. Governments play a role in monitoring and guaranteeing that HCNs allow for an allocation of medical resources that is fair and in accordance with acceptable principles of distributive justice. Nevertheless, within the framework or limits set by governments, there should be room for markets and civil society to operate. Whereas the whole of healthcare cannot be conceived of as a mere market (due to concerns highlighted above), HCNs have to face an economic reality and some degree of market thinking could help make them sustainable economic entities. A crucial role should also be played by civil society, as HCNs should not only be fair, just or economically sustainable, but also value-based. Within civil society health care institutions can come together around shared beliefs, values, interests and ideologies, thereby providing HCNs with the necessary value-based content. In short, governments can make HCNs just, markets can make them sustainable and civil society can make then value-based.

However, when the balance is skewed, there are severe ethical risks and HCNs might in effect lose their ethical justification. As a purely governmental/political construct HCNs might become out of tune with economic reality or might unduly enforce a particular value. Market and civil society coordination mechanisms keep this risk in check. Likewise, solely market driven HCNs might become unjust (e.g. by only providing care to those who can afford to pay or travel) or they might crowd out non-economic ideologies or values. Finally, civil society might fail to provide an overarching perspective, thereby potentially compromising the justice of the overall health system.

In short, whereas each of the coordination mechanisms has its ethical perspective, no perspective in itself justifies HCNs. What makes them ethical, is the correct balance between them.

## Footnotes

We propose three requirements rather than four as, we believe, the classic principles of non-maleficence and beneficence could be combined to form a single ethical requirement.

## Author Informations

**Kasper Raus** is a researcher at the Strategic Policy Cell of Ghent University Hospital and also affiliated with the Department of Philosophy and Moral Sciences of Ghent University. Currently his work focuses on ethical aspects related to interorganizational collaboration, health care networks and integrated care.

**Eric Mortier** is CEO of Ghent University Hospital and Professor of Anaesthesiology at Ghent University.

**Kristof Eeckloo** is head of the Strategic Policy Cell at Ghent University Hospital and Professor of Healthcare Management at the Department of Public Health of Ghent University. His research focuses on such issues as quality of care, patient participation and hospital governance.
